# New insights into the karyotype evolution of the genus *Gampsocleis* (Orthoptera, Tettigoniinae, Gampsocleidini)

**DOI:** 10.3897/CompCytogen.v12i4.29574

**Published:** 2018-12-19

**Authors:** Maciej Kociński, Beata Grzywacz, Dragan Chobanov, Elżbieta archałowska-Śliwa

**Affiliations:** 1 Institute of Systematics and Evolution of Animals, Polish Academy of Sciences, Sławkowska 17, 31-016 Kraków, Poland Institute of Systematics and Evolution of Animals, Polish Academy of Sciences Kraków Poland; 2 Institute of Biodiversity and Ecosystem Research, Bulgarian Academy of Sciences, 1 Tsar Osvoboditel Boul., 1000 Sofia, Bulgaria Institute of Biodiversity and Ecosystem Research, Bulgarian Academy of Sciences Sofia Bulgaria

**Keywords:** Orthoptera, *
Gampsocleis
*, chromosome evolution, FISH, 18S rDNA, telomeric repeats, Ag-NOR, fluorochrome staining

## Abstract

Five species belonging to the genus *Gampsocleis* Fieber, 1852 were analyzed using fluorescence *in situ* hybridization (FISH) with 18S rDNA and telomeric probes, as well as C-banding, DAPI/CMA_3_ staining and silver impregnation. The studied species showed two distinct karyotypes, with 2n = 31 (male) and 2n = 23 (male) chromosomes. The drastic reduction in chromosome number observed in the latter case suggests multiple translocations and fusions as the main responsible that occurred during chromosome evolution. Two groups of rDNA distribution were found in *Gampsocleis* representatives analyzed. Group 1, with a single large rDNA cluster on the medium-sized autosome found in four species, carried in the haploid karyotype. Group 2, represented only by *G.abbreviata*, was characterized by the presence of two rDNA signals. TTAGG telomeric repeats were found at the ends of chromosome arms as expected. The rDNA clusters coincided with active NORs and GC-rich segments.

## Introduction

The *Gampsocleis* Fieber, 1852 belongs to Gampsocleidini Brunner von Wattenwyl, 1893, a relatively small tribe of Tettigoniinae Krauss, 1902, which includes 17 currently recognized species and 7 subspecies mainly distributed in the Palearctic region (Cigliano et al. 2018). The taxonomic status of some taxa is still confusing and difficult to interpret. Molecular phylogenetic studies on *Gampsocleis* have also shown the taxonomic problem (Zhang et al. 2011, Zhou et al. 2011). In this paper, we focus on molecular and classical cytogenetics, providing data on the karyotype structure and evolution of the group.

Changes in chromosome number (karyotype variability) or structure can contribute to speciation (e.g. Dion-Côté et al. 2017; Gould et al. 2017). Information on cytogenetic markers is therefore useful for understanding the chromosomal organization and assessing the karyotype diversity of organisms. In this sense, chromosome rearrangements, such as Robertsonian fusions and inversions, can be important in tettigoniid karyotype evolution and also could have a role as drivers in the speciation process (Warchałowska-Śliwa 1998).

The chromosome number (2n) and fundamental number (FN = numbers of chromosome arms) have been described for more than 110 species from 37 genera of Tettigoniinae. Most Palaearctic species have a diploid number of 31 (male) and 32 (female) acrocentric chromosomes with an X0/XX sex chromosome determination system. This karyotype has been suggested to be ancestral/modal for most tettigoniids (White 1973, Warchałowska-Śliwa 1998). The genus *Gampsocleis* is an interesting group with diverse chromosome numbers. So far, eight species are cytogenetically known (reviewed in Warchałowska-Śliwa 1998). Two different karyotypes have been characterized in *Gampsocleis* 31 (FN = 31) and 23 chromosome (FN = 36) karyotype in the male. However, the knowledge of cytogenetic patterns is still fragmentary (Warchałowska-Śliwa et al. 1992).

The present study reports the chromosomal characters of five *Gampsocleis* species using both molecular fluorescent *in situ* hybridization (FISH), and conventional methods. These data are an initial step towards better understanding of the evolutionary relationships within this genus.

## Material and methods

A total of 18 specimens (adults and nymphs) belonging to five *Gampsocleis* species collected over several years (1990–2016) were selected for the study (Table 1). Gonads were excised and incubated in a hypotonic solution (0.9% sodium citrate), fixed in Carnoy’s solution (ethanol: acetic acid – 3:1) and then stored at +2 °C until use. The fixed material was squashed in 45% acetic acid. Cover slips were removed using the dry ice procedure, and the preparations were then air-dried.

**Table 1.** Localities of taxa, comparison of chromosome number and chromosomal localization of rDNA clusters, all forming active NOR.

Species	Collection sites and data	Geographical coordinates	No.	2n male	rDNA-FISH/ NOR
*Gampsocleisgratiosa* Brunner von Wattenwyl, 1862	China: Beijing area; 1995	no data	2	31	6
*Gampsocleissedakoviisedakovii* (Fischer von Waldheim, 1846)	Russia: Altai Mts, valley of Edigan River; 1998	51.1235N, 86.5149E	3	31	6
*Gampsocleisussurensis* Adelung, 1910	Korea: near Hamgyong Province, near Chongjin; 1990	41.79556N, 129.77583E	2	31	6
*Gampsocleisabbreviataebneri* Uvarov, 1921	(FYR) Macedonia: Sveti Nikola municipality, Bogoslovec ridge; 2008	41.78663N, 22.01893E	2	23	5, 8/9
*Gampsocleisabbreviatarenei* Miksic, 1973	Albania: Galichitsa Mts., above Pikina Voda place, above 1600 m; 2013	40.91136N, 20.85197E	1		
*Gampsocleisabbreviata* ssp.	Greece: Central Greece, Phthiotis, Palaiochori; 2015	38.70813N, 22.45736E	2		
*Gampsocleisglabra* Herbst, 1786	Bulgaria: Dobrich, Dobrich; 2006	43.60573N, 27.83478E	2	23	5
	Kazakhstan: (1) Aktobe, Safonowka, (2) Shimkent, Gavrilovka	42.20608N, 70.21833E	3		
	(3) Almaty, Uzunbylack; 2016	43.20317N, 78.98846E	1		

Fluorescence *in situ* hybridization (FISH) was performed as described by Grzywacz et al. (2018). The 18S rDNA probe was amplified with the 18S forward (5'-ACA AGG GGC ACG GAC GTA ATC AAC -3') and 18S reverse (5'- CGA TAC GCG AAT GGC TCA AT -3') primers (Grozeva et al. 2011). The primers TTAGG_F (5'- TAA CCT AAC CTA ACC TAA CCT AA-3'), and TTAGG_R (5'-GGT TAG GTT AGG TTA GGT TAG G-3') (Grozeva et al. 2011) were used for visualizing the telomeric DNA. The rDNA and telomeric probes were labeled using biotin-16-dUTP (Roche Diagnostics GmbH, Germany) and digoxigenin-11-dUTP (Roche, Diagnostics GmbH, Germany), respectively. The rDNA probe was detected with avidin-FITC (Invitrogen, USA) and the telomeric probe with anti-digoxigenin rhodamine (Roche Diagnostics GmbH, Germany). The chromosomes were analyzed under a Nikon Eclipse 400 microscope fitted with a CCD DS-U1 camera and NIS-Elements BR2.

The distribution of heterochromatin was revealed by C-banding techniques, as described by Sumner (1972) with a slight modification. In order to reveal the molecular composition of constitutive heterochromatin, some slides were stained with CMA_3_ to reveal GC-rich regions and DAPI to reveal AT-rich regions (Schweizer 1976). The silver staining of nucleolus organizer regions (NORs) was performed as previously reported in Warchałowska-Śliwa and Maryańska-Nadachowska (1992). At least 10 meiotic divisions (from diplotene to metaphase I) and three spermatogonial metaphases per male, and one to three males per species/population were analyzed using all the techniques. In all the analyzed species, the rDNA-FISH pattern, the locations of active NORs and heterochromatin pattern were recorded for meiotic bivalents in prophase I in the same individuals.

## Results

**Figure 1.**
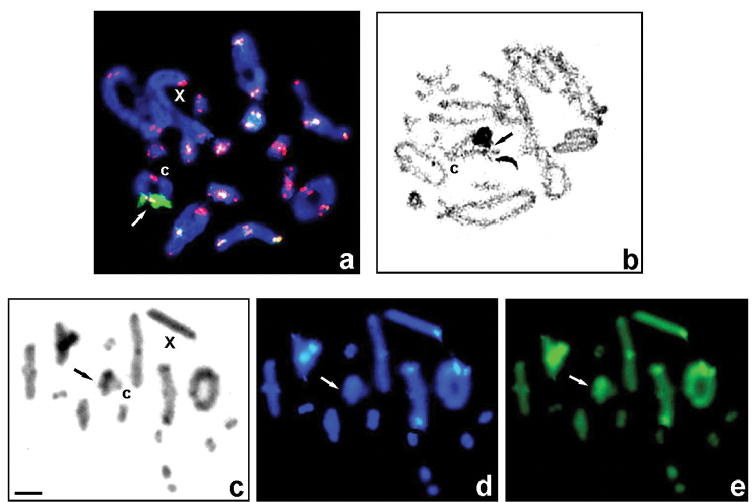
Examples of *Gampsocleis* species with 2n = 31 chromosomes (male): *G.s.sedakovii* (**a, c–e**) and *G.ussuriensis* (**b**) studied using different techniques: FISH with both 18S rDNA (green) and telomeric TTAGG (red) probes (**a**) in diakinesis revealed a single rDNA locus located distally on the 6^th^ bivalent (white arrow) and one active NOR visualized by AgNO_3_ staining (**b**) in diplotene (black arrow). C-banding (**c**) as well as fluorochrome staining of heterochromatin with DAPI (blue) and CMA_3_ (green) bands in diakinesis (**d** and **e**, respectively); black arrows indicate a C-band, and white arrows indicate very weak DAPI+ and bright CMA_3_+ signals located in a distal region on the 6^th^ bivalent. C **(a–c**), centromere; X (**c–e**), sex chromosome. Scale bar: 10 µm.

**Figure 2.**
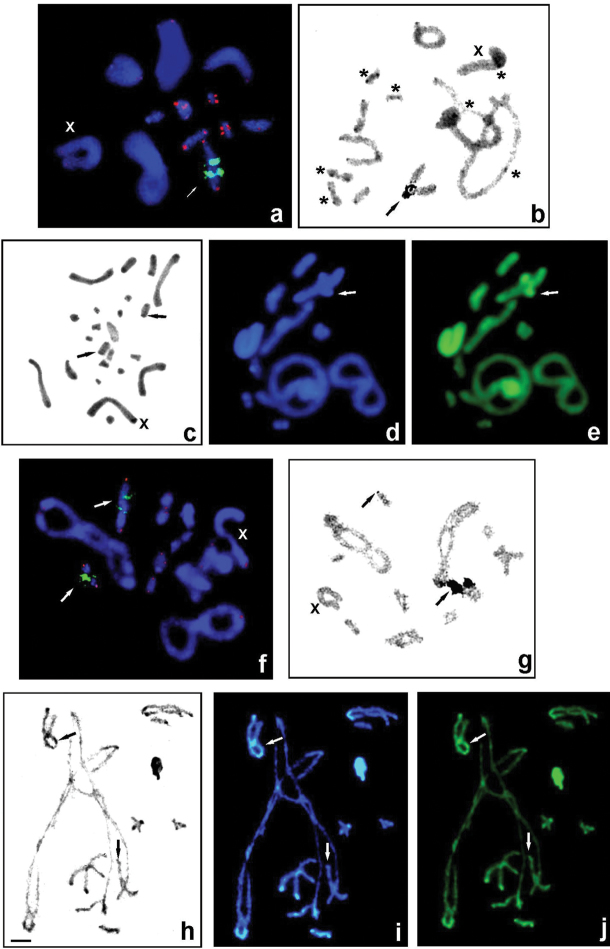
*Gampsocleis* species with 2n = 23 chromosomes (male): *G.glabra* (**a–e**) and *G.abbreviata* (**f–j**) studied using different techniques: FISH using 18S rDNA (green) and telomeric TTAGG (red) probes (**a, f**) and silver staining in diakinesis (**b,g**), C-banding of spermatogonial metaphase (**c**) and diplotene (**h**), and fluorochrome staining of heterochromatin with DAPI (blue) and CMA_3_ (green) (**d, i, e, j**). Arrows indicate rDNA clusters located near the telomeric region on the 5^th^ bivalent (**a, f**) and in a telomeric position on the short bivalent (**f**); active NORs co-localized with rDNA (**b, g**, black arrows); thin C-bands (**c, h**, black arrows) and weak DAPI+ (**d, i**, white arrows) and bright CMA_3_+ signals located near the telomeric region on the medium-sized bivalent (**e, j**, white arrows) as well DAPI-/CMA_3_+ signals on the telomeric region of the short bivalent (**i,j**, white arrows). Bi-armed chromosomes are marked by asterisks (**b**). X, sex chromosome. Scale bar: 10 µm.

We observed two different karyotypes with the sex determination system X0 in males of five species of the genus *Gampsocleis* (Table 1). The standard karyotype of *G.gratiosa*, *G.sedakoviisedakovii* and *G.ussuriensis* was characterized by a chromosome number of 2n = 31. In this case all chromosomes were acrocentric, consisting of four long, three medium and eight short pairs; the X chromosome was the largest element (Fig. 1a–e). In the second karyotype of *G.abbreviata* and *G.glabra* the chromosome number was reduced to 2n = 23 (Fig. 2a–j) with 11 pairs of autosomes arranged into three groups, 2 large, 3 medium, and 6 short pairs; among them, six pairs and the X chromosome were biarmed (Fig. 2b, marked with an asterisks). In both karyotypes, minor differences in the length of the short pairs of chromosomes sometimes made their precise identification difficult.

The localization of 18S rDNA in *Gampsocleis* was revealed by FISH and its activity analyzed by silver impregnation is summarized in Table 1. In four species we detected a single large rDNA cluster (per haploid genome) on a medium-sized autosome. This was evident distally/terminally to the centromere on the 6^th^ bivalent in males of *G.graciosa*, *G.s.sedakovii*, and *G.ussuriensis* (2n = 31) (Fig. 1a) or subterminally/subdistally on the 5^th^ bivalent in male individuals from four localities of *G.glabra* (2n = 23) (Fig. 2a). In contrast, two FISH signals were detected subterminally and terminally on the 5^th^ and 8/9^th^ bivalents, respectively, in *G.abbreviata* males (2n = 23) (Fig. 2f). FISH with the (TTAGG)*_n_* probe (tDNA-FISH) localized the telomeric sequences to the ends of chromosomes of the analyzed species as expected; no hybridization signals of the probe were found in the centromere region of biarmed chromosomes in species with 23 chromosomes. Generally, FISH signals of the telomeric probe in species with 31 chromosomes were stronger than in those with 23 chromosomes (Figs 1a, 2a).

After both C-banding and DAPI/CMA_3_ double staining, chromosome regions in the analyzed species showed discrete quantitative and qualitative variation in their constitutive heterochromatin. In *G.s.sedakovii*, *G.s.obscura*, *G.glabra*, and *G.ussuriensis* paracentromeric C-bands was uniformly present in long and medium-sized chromosomes, distal and interstitial bands are found to vary in size between these species, as described previously (Warchałowska-Śliwa et al. 1992, Table 1) and as example Fig. 1c (present study). In karyotypes with 23 chromosomes in both species, interstitial small C-bands near the distal region were present in the 5^th^ pair (Fig. 2c). Generally, paracentromeric thin C-bands on most of the autosomes were very weakly DAPI-positive (DAPI+) and CMA_3_-positive (CMA_3_+), whereas the thick paracentromeric C-bands showed bright homogenous DAPI+ (AT-rich) and bright CMA_3_+ (GC-rich) signals in some of the large and medium-sized autosomes and the X chromosome (Figs 1c–e; 2c–e, h–j). In addition, all species revealed weak C/DAPI+ and bright CMA_3_+ signals in the distal/subdistal region of a medium-sized bivalent (6^th^ or 5^th^) (Figs 1d,e; 2d,e,i,j). Additionally, in one short bivalent of *G.abbreviata*, a thin C-band in the telomeric region was visualized with the DAPI-/CMA_3_+ signal (Fig. 2i,j). Thus, the heterochromatin composition in these chromosomes exhibits distinct GC-rich bands coincident with active NORs and rDNA-FISH signals (Figs 1a,b,e; 2 a,b,e,g,j).

## Discussion

Our results are in line with previous studies (for a review see Warchałowska-Śliwa 1998), which revealed the advanced karyotype evolution in the genus *Gampsocleis*. The ancestral chromosome number 2n = 31 (FN=31) in Asian species was reported for males of two subspecies of *G.sedakovii* (*G.s.sedakovii*, *G.s.obscura*), *G.ussuriensis* and *G.gratiosa* (Hareyama 1932, Ueshima 1986, Kim et al. 1987, Warchałowska-Śliwa et al. 1992, Zhang et al. 2011), and for *G.buergeri* (Hareyama 1932). Only in *G.ryukyuensis* a metacentric X chromosome was observed (Ueshima 1986); in this case (FN=32), a pericentric inversion modified the centromere position, changing the morphology of the modal acrocentric sex chromosome to a biarmed X chromosome. Two Eurasian species, *G.glabra* and *G.abbreviata* (Warchałowska-Śliwa 1984, Warchałowska-Śliwa et al. 1992, present study), have reduced the chromosome number to 2n = 23 (FN = 36). This karyotype is probably the result of multiple translocations and fusions that occurred during the chromosome evolution in these species, as was suggested by Warchałowska-Śliwa (1984) and Warchałowska-Śliwa et al. (1992). In the last work, authors challenge the taxonomic status of *G.glabra* based on cytogenetic evidence (i.e. chromosome number). Currently, the Orthoptera Species File (Cigliano et al. 2018) include this species within *Gampsocleis*, based on morphological evidence.

In cytogenetic studies, the application of a variety of staining methods (classical and molecular) generally enables a better characterization of tettigoniid karyotypes and identification of genus/species-specific patterns (Grzywacz et al. 2017, Warchałowska-Śliwa et al. 2017). In this study, information revealed by FISH (rDNA and tDNA) is the first antecedent in species of *Gampsocleis*. Present result and previous cytogenetic data helps to interpret the chromosome evolution in this group. According to differences in the number and location of 18S rDNA signals, two groups were specified within the genus. The taxa belonging to group I were characterized by rDNA signals on one rDNA cluster in four species – *G.gratiosa*, *G.s.sedakovii*, *G.glabra*, and *G.ussuriensis*, while, in group II two rDNA loci in *G.abbreviata*. The karyotypes of three species (2n = 31) described both in this paper and previous work (Warchałowska-Śliwa et al. 1992), have a single active NOR and rDNA cluster on a medium sized autosome, probably M_6_, near the distal region. This localization suggests the occurrence of the same chromosome reorganization in the karyotype of the latter two species (2n = 23), whose evolution is difficult to explain. The presence of a distally located active NOR in only a single middle-sized bivalent has also been described in others European Tettigoniinae (Warchałowska-Śliwa et al. 2005). In most cases, a single 18S rDNA cluster/NOR is located near the paracentromeric/interstitial region within the subfamily (Grzywacz et al. 2017, Warchałowska-Śliwa et al. 2017), as in other tettigoniids (e.g. Warchałowska-Śliwa et al. 2013). Two rDNA/NOR loci restricted to subdistal/distal regions on different chromosome pairs (M_5_ and S_8/9_) were found in *G.abbreviata*. However, this difference between species with 2n=23 must be confirmed by analyzing a larger number of individuals to clarify whether it is a specific marker for *G.abbreviata*. The occurrence of TTAGG telomeric repeats was detected at chromosome ends in all the *Gampsocleis* species. This telomeric motif plays an important role in karyotype stability and is a common trait in insects (Vítková et al. 2005). Some interspecific differences in signal intensity may have been due to the presence of different numbers of telomeric repeats, whereas the lack of these sequences in the centromere region of the bi-armed chromosomes of *G.glabra* and *G.abbreviata*, which originated by chromosome fusion, is probably due to the loss of telomeric repeats during karyotype evolution (e.g. Warchałowska-Śliwa et al. 2013, 2017).

Discrete quantitative and qualitative differences in constitutive heterochromatin were discovered in the chromosomes of the analyzed species after both C-banding and DAPI/CMA_3_ double staining. The constitutive heterochromatin of all species analyzed was located in the paracentromeric and distal regions in some chromosomes and differed in size between species; similar observations were reported in previous studies of Gampsocleidini (Warchałowska-Śliwa et al. 1992) and other Tettigoniinae (e.g. Grzywacz et al. 2017, Warchałowska-Śliwa et al. 2017). DAPI and CMA_3_ staining showed very weak DAPI-positive (DAPI+) and CMA_3_-positive (CMA_3_+) segments. The thick C-bands coincided with bright homogenous DAPI+ (AT-rich) and bright CMA_3_+ (GC-rich) signals in the distal regions of the large and medium-sized autosomes, as well as in the paracentromeric region of the X chromosome. The presence of weak C/DAPI+ and bright CMA_3_+ signals near the distal region of a medium-sized bivalent is common for all *Gampsocleis* species, even in those with different chromosome numbers in their karyotype. The DAPI-/CMA_3_+ signal was only found in one short bivalent of *G.abbreviata* in a thin distal C-band. Generally, the position of the major rDNA sites in the currently analyzed species corresponds to the active Ag-NOR sites and some GC-rich bands.

Previous data (Warchałowska-Śliwa et al. 1992) threw light on the problematic taxonomic status of *G.glabra*, which was found to differ from the other examined species in this genus on its chromosome number. This is in agreement with the present results, which confirmed the chromosome number of *G.glabra* and showed similar results for *G.abbreviata*. These findings suggest important genetic differences between species between Eastern/Central Asia and Europe. However, there are a number of taxa in Western Asia (25% of all described *Gampsocleis* species) that have not yet been studied.

Species of *Gampsocleis* can be assigned into two groups distinguished by both the chromosome number and geographic range, in accordance with previous studies (Hareyama 1932, Warchałowska-Śliwa 1984, Warchałowska-Śliwa et al. 1992, Ueshima 1986, Kim et al. 1987). Geography plays an important role in generating genetic diversity. Our and previously published (Warchałowska-Śliwa et al. 1992) data suggest that *G.glabra* and *G.abbreviata* should be considered as belonging to a separate group. This is justified on the basis of their significant karyotype differentiation and could be either confirmed or rejected in future detailed genetic, morphological and/or behavioral studies. Further analyses on inclusive taxonomic sample of *Gampsocleis* may refine generic and intrageneric classification.

In conclusion, the present study offers new insights into the karyotype characteristics of bushcrickets that may be useful for interpret or understand relationships within the genus *Gampsocleis* as well as the subfamily Tettigoniinae. Changes observed in karyotypes may probably also play an important role in speciation. Additional species and methods (morphological and genetic characters) should be examined in order to further elucidate the relationships within the genus *Gampsocleis* and the tribe Gampsocleidini.
